# A Novel Heterozygous *De Novo MORC2* Missense Variant Causes an Early Onset and Severe Neurodevelopmental Disorder

**DOI:** 10.1155/2024/5906936

**Published:** 2024-01-02

**Authors:** Daniel Arbide, Nour Elkhateeb, Ewa Goljan, Carolina Perez Gonzalez, Anna Maw, Soo-Mi Park

**Affiliations:** ^1^Edinburgh Medical School, University of Edinburgh, Edinburgh, UK; ^2^Department of Clinical Genetics, Cambridge University Hospitals NHS Foundation Trust, Cambridge, UK; ^3^Exeter Genomic Laboratory Hub, Royal Devon and Exeter NHS Foundation Trust, Exeter, UK; ^4^Department of Paediatric Palliative Care, Cambridge University Hospitals NHS Foundation Trust, Cambridge, UK; ^5^Department of Paediatric Neurology, Cambridge University Hospitals NHS Foundation Trust, Cambridge, UK

## Abstract

Microrchidia CW-type zinc finger protein 2 (MORC2) is an ATPase-containing nuclear protein which regulates transcription through chromatin remodelling and epigenetic silencing. *MORC2* may have a role in the development of neurones, and dominant variants in this gene have recently been linked with disorders including Charcot-Marie-Tooth type 2Z disease, spinal muscular atrophy and, more recently, a neurodevelopmental syndrome consisting of developmental delay, impaired growth, dysmorphic facies, and axonal neuropathy (DIGFAN), presenting with hypotonia, microcephaly, brain atrophy, intellectual disability, hearing loss, faltering growth, and craniofacial dysmorphism. Notably, variants in *MORC2* have shown clinical features overlapping with those of Cockayne and Leigh syndromes. Here, we report a case of *MORC2*-related DIGFAN syndrome in a female infant caused by a novel heterozygous *de novo* variant. The condition was early onset and severe, further expanding the range of genotypes associated with this disorder. Clinical features included unilateral hearing loss, developmental delay and regression within the first year of life, microcephaly, severe feeding difficulties, and faltering growth, resulting in death at 13 months of age.

## 1. Introduction

Microrchidia CW-type zinc finger protein 2 (*MORC2*, MIM: 616661) encodes a DNA-dependent ATPase-containing protein expressed in all tissues, with enrichment in the brain. It is composed of a catalytic ATPase domain composed of the GHKL-type domain and the S5 domain, three coil-coiled domains allowing dimerisation or protein complex interaction, a zinc-finger CW domain allowing DNA interaction, and a CHROMO-like (CHRomatin Organisation MOdifier) domain allowing histone recognition. It regulates transcription through human silencing hub (HUSH)-mediated chromatin remodelling and epigenetic modification [1, 2]. In 2016, it was identified as a newly causative gene for dominantly inherited Charcot-Marie-Tooth (CMT) Type 2Z disease [3]. *MORC2*-related CMT2Z displays high intrafamiliar variability in symptom onset and severity, ranging from rapidly progressing and early onset within the first decade to slowly progressing and late onset [4].

Ever since then, other studies have consolidated the association with CMT2Z [5, 6], while further widening the scope of *MORC2*-linked disorders to include a complex and multisystemic neurodevelopmental syndrome consisting of developmental delay, impaired growth, dysmorphic facies, and axonal neuropathy (DIGFAN; MIM 619090), presenting with hypotonia, microcephaly, brain atrophy, intellectual disability, hearing loss, faltering growth, and craniofacial dysmorphism [7–9]. In addition, *MORC2*-related disorders have exhibited phenotypical characteristics overlapping with those of Cockayne and Leigh syndromes [7, 10–12]. A literature review of *MORC2* variants identified a wide variety of other associated clinical manifestations, including precocious and delayed puberty, hypothyroidism, strabismus, hirsutism, cataracts, hearing loss, hammertoes, frequent respiratory infections, growth hormone deficiency, vitamin D deficiency, and increased prolactin [12].

Because of its association with neurological disorders, *MORC2* has been suggested to have a role in neuronal development and survival. It was found to be highly expressed in human embryonic and adult neural tissues and dynamically regulated in the developing and mature murine nervous systems [13]. Functional assays have found that *MORC2* variants affect neurone survival, apoptosis, neurite outgrowth, and abnormal axon morphology in keeping with the axonal neuropathy seen in patients with *MORC2*-related disorders [13, 14]. Furthermore, *MORC2* may have a role in tumorigenesis, promoting the proliferation, invasion, and migration of glioma by inactivation of *PTEN/PI3K/AKT* signaling [15].

We report the case of a severe and early lethal *MORC2*-related disorder with a novel pathogenic *de novo* variant, encompassing history, clinical features, progression, subsequent investigations, and multidisciplinary management, including paediatric palliative care, demonstrating the disorder at the severe end of the phenotypic spectrum.

## 2. Clinical Report

The patient, a female infant presenting with developmental delay and regression with severe feeding difficulties, was initially referred to paediatrics by her GP at 11 months of age and was subsequently seen by paediatric neurology and clinical genetics. She was born to an unrelated and healthy White British couple, with a healthy 8-year-old half-brother on the father's side from a previous relationship.

The patient was born at 39 weeks via normal vaginal delivery with no complications. Early in the pregnancy, the mother suffered from hyperemesis gravidarum, and from 36 weeks, she took oral acyclovir as prophylaxis against herpes simplex. Scans showed placenta praevia, and while these showed no obvious concerns regarding the baby, some concerns relating to foetal movements were noted in the later stages of pregnancy. At birth, the baby weighed 3.42 kg (near the 75^th^ centile) with a head circumference of 32.7 cm (near the 25^th^ centile). Talipes was noted in the left foot, and a referral to physiotherapy was arranged. Shortly after birth, the parents noticed the baby to be generally sleepy; she did not cry much or demand feeds, necessitating timed feeds with an alarm. Although no seizures were reported in the neonatal period, the mother reported abnormal hand movements in the first 4 months.

Early development was encouraging, as she was able to roll both ways by 5 months and sit without support for brief periods by 6 months. It was noted, however, that she tended to take weight on her tiptoes when held upright, never bounced, and was not happy lying on her front or able to push herself up on her arms. Parental concerns first arose at 6 months of age, as the patient was no longer meeting developmental milestones. She did not progress beyond brief periods of sitting up, was not able to tolerate lumpy food when weaning, and was found to have unilateral hearing loss, which underwent investigation.

She was reviewed by the paediatric neurology and clinical genetics team when she was 51 weeks old. By this point, the mother reported a clear history of developmental regression and loss of skills, particularly in the past 4 weeks, with the patient no longer being able to roll over or sit even with support. She showed left-sided preference, moving her left arm and leg more than the right, as well as limited head control. She had a palmar grasp but no pincer grip, no stranger anxiety, and her language consisted of babble only. She had also been having increasing difficulties with feeding and weight loss and was frequently choking and gagging, necessitating the insertion of a nasogastric tube for feeding. In addition, she had developed constipation, requiring treatment with Movicol.

**Figure 1:**
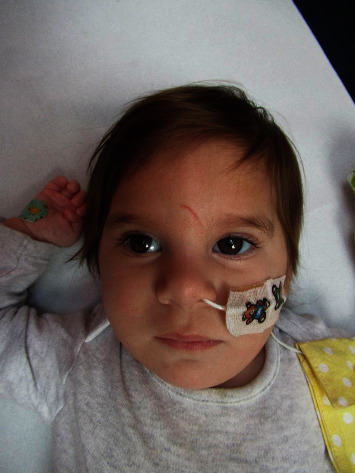
Frontal view of patient at 51 weeks of age displaying several distinctive features including almond-shaped eyes with epicanthic folds, mild ptosis bilaterally, widened nasal bridge, mild brachydactyly in her feet, and a degree of micrognathia (Figure 1).

On examination, she now had a head circumference of 41.5 cm (less than the 1^st^ centile, −2.46 SD) and weighed 6.89 kg (2^nd^ centile). She displayed several distinctive features including almond-shaped eyes with epicanthic folds, mild ptosis bilaterally, a widened nasal bridge, mild brachydactyly in her feet, and a degree of micrognathia (Figure 1). She had a nasogastric tube in situ. Her tone was slightly reduced in all 4 limbs with brisk reflexes detected in the lower limbs, and bilateral striatal large toe was noted in addition to poorly coordinated, jerky movements. No peripheral neuropathy was found on examination, and nerve conduction studies were not performed due to the rapid progression of the patient.

**Figure 2:**
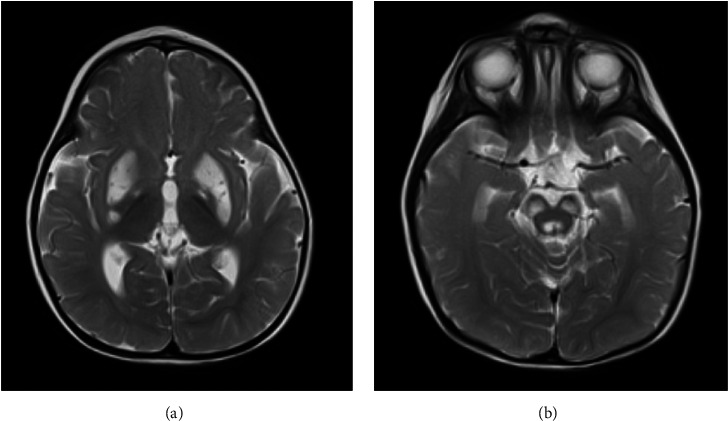
Brain MRI in the patient at the age of 51 weeks. (a) Axial, T2-weighted images showing bilateral high signal intensity in the lentiform nuclei, putamen, and globus pallidus. (b) Axial, T2-weighted images showing bilateral high signal intensity in the cerebral peduncles and midbrain.

Cerebrospinal fluid (CSF) and serum analysis showed significantly raised lactate levels of 5.1 mmol/L (reference range 1.1–2.4 mmol/L) and 4.7 mmol/L (reference range 0.6–2.5 mmol/L), respectively. Brain imaging with MRI revealed bilateral and symmetrical high intensity on T2 with restricted diffusion on diffusion-weighted imaging in the lentiform nuclei, putamen, and globus pallidus. There was a slight enlargement of the ventricles, suggestive of early volume loss, and evidence of change in the cerebral peduncles and midbrain (Figure 2).

Given the investigation results and clinical features, a neurometabolic condition with an underlying genetic cause was thought to be most likely. Rapid trio whole exome sequencing (R14), mitochondrial genome analysis, and CGH array were therefore undertaken. Exome sequencing revealed the presence of a heterozygous, *de novo* pathogenic missense variant in the *MORC2* gene, NM_001303256.3: c.262G > C p. (Ala88Pro). Although this was a novel variant, other distinct missense variants affecting the same and adjacent residues (positions 88 and 87) have been reported as pathogenic in the literature, both clinically with the presence of CMT and in functional assays [7]. Application of the January 2018 ClinGen SVI Bayesian classification framework to the variant seen in the patient showed the posterior probability to be 0.999, classifying the variant as unambiguously pathogenic. The presence of this variant in the context of the displayed clinical features established the diagnosis of a *MORC2*-related neurodevelopmental disorder. Microarray analysis showed a 14 Kb heterozygous interstitial loss of 12q21.31 of unclear significance, with a dosage sensitivity score of −1.73 and a sampling probability [16] of >5%, denoting a low chance of pathogenicity. Mitochondrial genome analysis of blood cells identified no known or likely pathogenic variants following sequencing, diminishing the likelihood of a coexisting mitochondrial disorder.

Over the next month, the patient's health continued to deteriorate significantly, with regular episodes of choking, gagging and respiratory distress, and further regression of motor skills with increasing fatigue. The parents found it increasingly challenging to meet her care requirements, at times needing to support and maintain her airway; after discussion with the paediatric neurology and palliative care teams, they decided to focus on optimising quality of life with supportive hospice care. The patient subsequently died at 13 months of age.

## 3. Molecular Analysis

Trio rapid exome sequencing of genomic DNA extracted from peripheral blood samples from the proband and their parents was performed using the Twist Human Core Exome Kit (Twist Bioscience, San Francisco, CA, USA) following the manufacturer's protocols https://www.twistbioscience.com/resources/protocol/library-preparation-twist-umi-adapter-system. Briefly, DNA was quantified on the Qubit fluorometer (Thermo Fisher Scientific, Massachusetts, USA) fragmented enzymatically, end-repaired, and dA-tailed. Indexed with Twist UMI Adapters and amplified using PCR Amplify Twist UDI Primers. Indexed libraries were pooled, and paired-end reads of 150 bp were sequenced on the NextSeq500 (Illumina, San Diego, CA, USA). De-multiplexing and alignment of the FASTQ reads to the reference genome (GRCh37/Hg19) were done using BWA-MEM (v0.7.12) 7, and GATK (v3.4-46) 3 was used for variant calling and quality filtering. Subsequently, Alamut Batch (Interactive Biosoftware, Rouen, France) was run to perform variant annotation. Variant filtering was carried out as previously described (Lango et al. 2011 https://www.ncbi.nlm.nih.gov/pmc/articles/PMC4062962/).

Gene-agnostic trio bioinformatics pipeline was used for analysis and variant prioritisation. Orthogonal validation was performed by targeted Sanger sequencing of *MORC2* in all three family members.

## 4. Discussion

Our patient has a confirmed *MORC2*-related neurodevelopmental disorder, arising from a *de novo*, germline heterozygous pathogenic variant. Pathogenic variants in *MORC2* have been associated with CMT disease since 2016 [3], although this case more closely resembles that of another *MORC2*-related disorder, a complex neurodevelopmental syndrome characterised by developmental delay, impaired growth, dysmorphic facies, and axonal neuropathy (DIGFAN; MIM 619090) [7]. The variant we report is novel and located where other missense pathogenic variants affecting the same and adjacent residues (positions 88 and 87) have also been reported on ClinVar and in the published literature [7, 17]. This locus within the ATPase catalytic domain appears to be a hot spot for recurrent pathogenic variants in *MORC2*, leading to conditions such as CMT2Z and DIGFAN syndrome. Biochemical studies of *MORC2* have suggested that disease-causing mutations at this locus may interrupt crucial ATPase dimerisation dynamics, in turn modulating epigenetic silencing by the HUSH complex [18]. Five individuals harbouring the c.260C > T (p.Ser87Leu) variant all presented with significant neurological symptoms at a young age, including gross motor delay and hypotonia [7], perhaps indicating that pathogenic variants at this genetic locus confer a particularly severe phenotype. This is consistent with the clinical picture seen in our case and may be of diagnostic and prognostic value when variants are detected in this locus.

Because plasma and CSF samples demonstrated an increased level of lactate, a mitochondrial cause for this neurometabolic disorder, such as in the Leigh syndrome, was also suspected but was excluded using mitochondrial genome sequencing. In a separate case, a 27-month-old child with *MORC2*-related CMT2Z presented with developmental lag, dysphagia, and fatigue, with elevated lactate and MRI lesions reminiscent of those seen in the Leigh syndrome [12]. Several other patients with *MORC2*-related DIGFAN syndrome were also suspected to have a mitochondrial disease and displayed Leigh syndrome-like lesions [7], highlighting the clinical similarities and diagnostic challenges in differentiating these conditions. Similarly, a phenotypic overlap between the *MORC2*-related neurodevelopmental disorder and Cockayne syndrome has been described [10, 11]. Pathogenic *MORC2* variants were uncovered in several individuals with genetically unsolved, clinically diagnosed Cockayne syndrome. Conversely, patients with confirmed *MORC2* variants were noted to exhibit features evocative of the Cockayne syndrome. It is therefore important to recognise both the range of differential diagnoses presenting with this constellation of symptoms, as well as the clinical heterogeneity, as described above, associated with *MORC2* variants, highlighting the value of early genetic testing in patients presenting with these phenotypes.

In conclusion, we describe a novel pathogenic variant within the ATPase catalytic domain of *MORC2*, causing a neurodevelopmental disorder with a severe, early-onset, and ultimately fatal clinical course, adding to the expanding range of genotype-phenotype relationships in patients with *MORC2*-related disorders. Our case also flags the diagnostic challenges inherent in early-onset severe paediatric neurodevelopmental disorders where there is a clinical overlap with other related disorders. Rapid agnostic whole exome or genome sequencing has a significant chance of achieving an early diagnosis which can differentiate treatable conditions from those that are not. In the latter, it can also help guide further medical management, including palliative care to ensure optimal symptom management, advance care planning discussions, and end-of-life care wherever appropriate (end-of-life care for infants, children, and young people with life-limiting conditions: planning and management https://www.nice.org.uk/guidance/ng61).

## Data Availability

Data are not available as no new data were created or analysed in this study.
